# Medical Extracts

**Published:** 1885-06

**Authors:** 


### Medical Extracts.

### Erysipelas and the Antiseptic Method.

M. Vermeuil, in a communication on " Erysipelas and the Antiseptic Method," directs attention to the fact that in the practice of surgery, where the antiseptic method is adopted, erysipelas, if it has not completely disappeared, has become extremely rare, has almost ceased to be epidemic and even endemic, being only worthy of the term sporadic.

The observations, of which he gives an analysis, lead to the following conclusions:

1. Erysipelas, infectious, contagious, auto-inoculable, has multitudinous origins, which it will be difficult to suppress yet awhile.

2. In our great centres, erysipelas, essentially endemic, has two distinct sources of supply—the one exterior, the town; the other interior, the hospital—mutually poisoning each other.

3. One has no direct control over the endemic of the town, nor on the sporadic cases of the hospital; nevertheless, one is much more powerful in the home centre. There one can to a large extent anticipate the appearance and extension of the mischief by minute precautions against auto-inoculation, by antiseptic dressings, by isolation, if this be practicable; and, in default of this, by creating about the patient a circumscribed antiseptic atmosphere.

4. The diminution of erysipelas in surgical wards results, not only in the healthiness of these wards themselves, but of the whole hospital and its immediate surroundings as well—healthiness most plainly demonstrated by the considerable diminution of erysipelas cases coming from without.

5. If the prophylactic and curative resources, of which science has plainly demonstrated the efficacy, were rigidly and generally applied in town and in hospital, it might be hoped that erysipelas would become as rare as pyæmia, and perhaps disappear as completely as hospital gangrene.—*Bulletin de l'Académie de Médicine*, No. 8, 1885.

### Abdominal Tumour.

M. Chauffard, in a case of abdominal tumour, believed to be kidney, adopted the following expedients in order to complete the diagnosis: (*a*) Aspiration on two occasions withdrew fluid of a urinous odour, and charged with pus, similar to that found in the urine. (*b*) Salicylate of soda (2 grammes) was administered, and on the following day the fluid obtained by aspiration was found to contain indications of its presence. (*c*) Fuchsine in solution was injected into the purulent cavity, and one hour afterwards the urine was found to be coloured red.

From these observations, the tumour in the left loin was believed to be a cystic pouch, containing urine and pus, communicating with the kidney on the one hand, and on the other with the bladder through the ureter. M. Chauffard concluded that the case was one of pyelo-nephritis, possibly dependent on a calculus partially obliterating the ureter. This diagnosis was verified by the result of the operation of nephrectomy, performed by M. Polaillon, and the patient made a speedy and complete recovery.—*Bulletin de l'Académie de Médicine*, No. 18, 1885.

### Acute Articular Rheumatism.

M. Birnheim presents to the Academy of Medicine of Paris a note on the efficacy of antipyrin in acute articular rheumatism. The drug was administered to ten patients, in doses of six to eight grammes per diem, in divided doses, at intervals of one hour. It was found to be of real advantage, and always to give rise to a disappearance, or notable diminution of the articular swelling. This drug may, therefore, take a definite place side by side with salicylate of soda: it has a very rapid anti-pyretic effect, and is better tolerated, having less tendency to provoke cerebral disturbance.—*Bulletin de l'Académie de Médicine*, No. 9, 1885.

### Pulmonary Tuberculosis in Families.

M. E. Leudet (de Rouen), in a paper on "Pulmonary Tuberculosis in Families," arrives at the following conclusions:

1. Families are divided into those which have presented only one case of tuberculosis, and those which have presented several.

2. Tuberculosis occurring only in one member of a family for several generations is said to be acquired. This acquired tuberculosis attacks more easily persons previously debilitated, either congenitally or by previous illness. Pneumonia and bronchitis do not seem to predispose to it in a more marked manner. Persons of the same families, free from tuberculosis, die at an advanced age of maladies other than those connected with the respiratory organs.

3. Hereditary transmission of phthisis exists in more than half the cases. Direct heredity of tuberculosis from fathers and mothers to children has been ascertained in 82 families. Heredity transmitted from father, mother, grandfather and grandmother, from uncle or aunt, to their descendants, existed in 108 families out of 214. In 106 families no trace of heredity was found. Hereditary transmission is more frequent in the maternal than in the paternal line. Pulmonary tuberculosis generally appears in the descendant at the age when the disease is most frequent—that is to say, from 13 to 35 years—whatever be the age at which phthisis manifested itself in the ancestor. Hereditary tuberculosis manifests itself at a less advanced age than acquired tuberculosis. The tubercular heredity of the two ancestors increases the chances of transmission to the descendant, as also if one of the tubercular descendants is united to a person come of a tuberculous family. In tuberculous families, one generation may escape the complaint, the other being attacked.

4. Pulmonary tuberculosis constitutes sometimes a morbid selection, that altogether exterminates *degenerated families.* That extension of the disease is encouraged in the ancestors by the existence of tuberculous cachectic affections, general paralysis, madness, idiocy, &c. In the descendants it is explained by the frequency of cretaceous pulmonary tubercle in some; in others, by the arrests of development, idiocy, hæmophilia, deafness, internal otitis, coxalgia, infantile paralysis.

5. Diseases of the bones and articulations are met with in 20 per cent. of tubercular families. Tubercular affections of bones or articulations oftener precede tuberculosis of the lung.

Auto-inoculation is more frequent during the suppuration of tubercular osteitis. Coxalgia, ozœna, otitis appear to be less serious, and more capable of cure. They exist alone in certain branches of contaminated families.

6. The marriage of a healthy subject, or of a subject springing from a healthy family, with a tuberculous subject, or the issue of a tuberculous subject, even during several generations, diminishes, but does not extinguish, the chances of tuberculosis in the descendant.

7. Does the propagation of tuberculosis by contagion exist in families? Fifty-five families, comprising 415 individuals, have presented only one tuberculous case; 88 families, comprising 1,070 individuals, have presented several tuberculous cases. Contagion is, therefore, not the rule.

8. Marital contagion is, at least, rather rare; it has only appeared possible in 7 couples out of 68. In 61 couples, one of the parties remained free from disease.

9. Contagion appears to derive support from this fact: that in 33 families, of which 15 were tainted with hereditary tuberculosis, 73 children out of 124 were affected with pulmonary tuberculosis in a period varying from one to nine years. More than half the children so affected were debilitated and of feeble constitution previous to the attacks.

10. The progress of pulmonary tuberculosis is much slower in the better classes than amongst the working population. The poor, whose tuberculosis is arrested, often die of consecutive visceral degenerative affections.

11. The rapidity or the slowness of tuberculosis has no connection with heredity.

12. The cure of pulmonary tuberculosis is to be met with in hereditary phthisis, as well as in the acquired disease. Tuberculosis can be cured at all periods.—*Bulletin de l'Académie de Médicine*, No. 15, 1885.

### On the Employment of Corrosive Sublimate in Otology.

Dr. E. Menière, in a paper read before the Société Française d'Otologie, in December, 1884, says that he finds the following mixture of phenic acid and glycerine has done him most service in the treatment of chronic otorrhœa, which, as everyone who has had much to do with aural disease knows, is often a most obstinate affection to treat:

| | |
|---|---|
| Crystallised phenic acid ... ... | 1 to 10 grammes |
| Price's glycerine ... ... ... ... | 10 " |

A small quantity of this is used after injections of warm water. He also alludes to what is a matter of every-day experience—that, in certain refractory cases, it is necessary to vary the remedies used; and he especially mentions, that although the alcohol treatment is often very successful, still it frequently has to be stopped, and then again taken up in the same case; with which opinion Dr. Baron thoroughly agrees, having found that where the alcohol treatment, after several weeks of continuous employment, has not been able to do more than to prevent granulations ftom growing, and has not been able to stop the discharge, nor to bring about a healthy condition of the meatus, the substitution of the dry boracic acid treatment frequently does very good service, but that even here the alcohol may have to be used again before cure is brought about.

Dr. Menière then goes on to say that he has used a mixture of corrosive sublimate and glycerine for some months past:

| | | |
|---|---|---|
| Price's glycerine | ... ... | 10 grammes |
| Corrosive sublimate ... | ... ·05; ·15; ·30 | " |

He lays great stress on the necessity of using *perfectly pure* glycerine, and considers Price's the best.

As a matter of experience, he has found that during the period of active sero-purulent secretion, the corrosive sublimate is not better in any way than the phenic acid; but, during the period of oozing, with or without perforation, it is very serviceable, and has produced rapid cures in a relatively short space of time. No inconvenience was noticed in its use.

His paper ends by stating that perseverance in the use of remedies, even after a state of dryness of the ear has been brought about, is very important, in order that it may be lasting.—*Revue Mensuelle de Laryngologie et d'Otologie*, April 1st, 1885.

### On the Amount of Pepsin contained in the Gastric Juice in Normal and Pathological Conditions of the Stomach.

Dr. E. Schütz, as the result of very careful examinations, comes to the following results: The gastric juice, obtained by means of a tube from the healthy empty stomach, contains, as a rule, pepsin, the amount of which varies very little in the same individual at different times; but in different individuals it varies between ·4 and 1·2 units. Where the stomach is diseased, the pepsin obtained, under similar conditions as in the healthy person, is much less, as a rule, and may even be altogether absent in severe cases, or those that have lasted for some time. In less severe cases there was also observed some diminution of the pepsin. In nervous dyspepsia there is little or no change in the amount of this substance present; also the same was noted in the dyspepsia of anæmic and chlorotic patients, and even in those suffering from tuberculosis. A normal or only slightly decreased amount of pepsin is almost always accompanied by a strongly acid reaction of the gastric juice, whilst if the amount is much decreased, or there is none at all present, the reaction is, as a rule, alkaline, neutral, or but feebly acid.—*Deutsch Mediz. Zeitung*, May 4th, 1885.

### Experiments on the Power of Milk taken from an Animal suffering from Splenic Fever, to Communicate the Disease to others.

Messrs. Chambrelent and Moussons, writing to the *Revue für Thierheilkaude*, June, 1884, on this important matter, give the results of some experiments made by them.

Feser of Munich, in the year 1879, said that the milk of an animal suffering from splenic fever contained the infectious substance, and the following experiments of Messrs. C. & M. confirm this statement:

1. A guinea-pig that had had a litter ten days previously was inoculated with the cultivated virus of anthrax, as a result of which it died in the course of a day. An hour after its death, a drop of milk was removed from it, and cultivations were made in beef bouillon. A guinea-pig that was inoculated with a drop of this cultivation fluid died after two days, and in its blood the bacillus anthracis was found. A second guinea-pig, which had been inoculated, died during the next day.

2. A similar experiment, using the same methods but not the same animals, resulted in the death of two guinea-pigs.

3. With the same virus as used in experiment No. 2, a rabbit, which was yielding milk at the time, was inoculated; but it remained healthy, and its milk did not produce anthrax bacilli on cultivation.—*Deutsche Mediz. Zeit.*, No. 56.

### On the Treatment of Parenchymatous Goitre.

Dr. Moritz Schmidt finds that large doses of iodide of potassium, combined with the constant application of cold, by means of Leiter's apparatus, is very useful in this affection, it having yielded good results in six cases. Where there is great difficulty of breathing, Dr. S. applied the cold continuously; in less severe cases, for three hours in the morning, and the same length of time in the evening, and not during the rest of the day.—*Deutsche Med. Zeit.*, No. 57.

**The Parasitic Origin of Cancer.**

The resemblance in many respects between cancer and tuberculosis has led of late to a renewed interest in the old question as to the infectiousness of cancer. Dr. A. Ledoux-Lebard, in the April number of the *Archives Génèrales de Médecine*, makes an elaborate plea in favour of the view that cancer is a parasitic disease. Under the head of clinical and etiological consideration, he shows the analogies between cancer and known chronic infectious diseases, like tuberculosis. Under the head of pathological anatomy, he argues that cancer is a specific inflammation ; while, under the head of experimental pathology, he cites the numerous experiments that have been made to determine the inoculability of cancer. The author admits that none of these are demonstrative, while most of them are absolute failures. The best cases are those of Goujon, who successfully inoculated cancerous nodules in a guinea-pig, using a fragment of an epithelial cancer from an animal of the same species. Klenke also is said to have successfully inoculated a horse with melanotic cancer. Dr. Lebard further cites facts which, as he thinks, prove that cancer is auto-inoculable. The natural history of actinomycosis is given as illustrating an infective parasitic disease, allied clinically to cancer, which is undoubtedly not inoculable.

Lebard has himself attempted inoculations of cancer without success. He has also tried, by various staining methods, to discover a characteristic micro-organism. In this, too, he failed, and at present the view that cancer is a parasitic disease continues to rest only upon analogical reasoning.—*N. Y. Med. Record.*

**Antipyrine.**

So much has already been written concerning this new drug that we cannot hope to add anything new. The excellent article by Dr. Draper, in the *Journal* for April 18th, covers in a short space the main facts with reference to antipyrine, both historical and clinical. He speaks highly of its antipyretic properties, but calls attention distinctly to the fact that it does not appear to have any specific effect upon the febrile affections in the treatment of which it is recommended. This is an important point, since the reader is apt to form a contrary impression after reading the enthusiastic reports of German observers. Dr. Draper adds the caution that the drug is one which cannot be given indiscriminately. Occasionally unpleasant symptoms are noticed, such as vomiting, chills, and profuse perspiration.

In connection with Dr. Draper's article, we would refer the reader to one on the same subject, in another number of the *Journal*, in which the writer, Dr. Butler, is equally emphatic in praise of the drug as a pure febrifuge. He notes especially the great rapidity with which the temperature was lowered, and makes the important statement that antipyrine is not an antiperiodic, in which fact lies one of its principal points of difference from quinine. Neither of these authors has attempted an explanation of the *modus operandi* of the drug; in fact, in the present state of our knowledge, any explanation would necessarily be largely theoretical. Dr. Draper expresses its action tersely when he says that "it smothers, but does not extinguish, the fire." It is still *sub judice*, and we trust that a sufficient number of observations will be made in hospital practice to fully test the new remedy. The one criticism to be urged against scientific medicine in this country is, that the observers are so few and far between. When some brilliant novelty (such as cocaine) appears, every-one is ready to try it; but for patient, long-continued experiments we are not distinguished. We hope to see antipyrine given a fair trial, not only by city practitioners, but by their brethren in the country, the latter of whom are frequently quite the equals of their metropolitan *confrères* as original observers.—*N. Y. Med. Journal*, May 9th, 1885.

### Paraldehyde as a Sleep-producer in the Treatment of the Insane.

As the production of sleep is often a chief factor in the treatment of the insane, I am prompted to offer the result in one hundred and fifty two cases of mental disease in which sleep was induced by a recent hypnotic which is now attracting much attention. When paraldehyde is given to man in moderate dose (fifty minims), the most marked result, in the majority of instances, is a quiet sleep of from two to seven hours, which is induced in ten to forty-five minutes after its absorption.

Its advantages over chloral in our experience are mainly that there is no danger from its action on the heart; in fact, one hundred minims have been given in cases of acute mania without the slightest noticeable effect upon the heart or respirations, the repose simulating closely natural sleep, the subject being easily aroused, but soon dropping off again when let alone. No convulsive effect or dreamy stimulation of the mind has been observed, its first effect being apparently upon the cerebral hemispheres. Never have we seen the awakening accompanied by any unpleasant symptoms; in fact, there is no objection on this score save the disagreeable odour of the breath, which is perceptible for from twelve to seventeen hours after the exhibition of the drug.

In cases of melancholia paraldehyde has acted commendably; even in patients who would sit for hours deploring their unhappy lot, and whose brains were filled with fears of an overwhelming calamity, a dose of sixty minims of the drug has calmed them into a quiet and apparently dreamless, refreshing sleep. In several instances, however, we found it necessary to increase the dose to seventy-five minims, but this amount uniformly brought on a most gratifying rest.

In those cases where paraldehyde is required to be given for a length of time, a certain tolerance would be expected; but the observations lead us to believe that the tolerance of paraldehyde does not manifest itself sooner than other hypnotics, and whether the increased dose was needful on account of a slight aggravation of the disease, or a tolerance of the drug, as yet we are unable to say: in all probability both have taken a part.

As an anodyne (in a limited number of trials) the remedy has failed either to relieve pain or produce sleep, although seventy-five minims of the drug have been taken; in no case, however, was nausea excited.

Our results having been so uniformly satisfactory, we consider that paraldehyde is a valuable addition to our list of hypnotics; and where a sleep-producer must be given for a length of time, we consider it a most efficient, safe, and reliable remedy, *the sleep produced being in proportion to the size of the dose administered.—Philadelphia Medical Times*, May 16th, 1885.

